# Response to ‘Feasibility of tailored treatment based on risk stratification in patients with early rheumatoid arthritis’

**DOI:** 10.1186/s13075-015-0680-8

**Published:** 2015-06-20

**Authors:** Nathan Vastesaeger, Bruno Fautrel, Josef Smolen

**Affiliations:** Department of Medical Affairs, MSD Denmark ApS, Lautrupbjerg 4, 2750 Ballerup, Denmark; UPMC-Paris 6 University, GRC 08, Pierre Louis Institute of Epidemiology and Public Health, 47-83 boulevard de l’Hôpital, 75013 Paris, France; AP-HP, Department of Rheumatology, Pitié Salpêtrière Hospital, 47-83 boulevard de l’Hôpital, 75013 Paris, France; Division of Rheumatology, Department of Medicine 3, Medical University of Vienna, Waehringer Guertel 18-20, A-1090 Vienna, Austria

Markusse and colleagues recently investigated whether rheumatoid arthritis patient subgroups formed according to the presence of poor prognostic factors respond differently to initial monotherapy or combination therapy [1]. Since both poor- and good-prognosis subgroups experienced a better response to initial combination therapy, the authors concluded that patient-tailored treatment based on prognosis as suggested by the European League Against Rheumatism (EULAR) recommendations [2] is currently not feasible.

As a general remark, the authors should be reminded that the EULAR recommendations primarily suggest combination of methotrexate with low-dose glucocorticoids because its efficacy is not surpassed by biologicals and it prevents overtreatment in 20 to 25 % of patients [3, 4]; delaying tumor necrosis factor-inhibitor initiation by 6 months does not affect outcomes [5]. Moreover, the definitions of poor prognosis (PP) used by Markusse and colleagues contrast with the stratification suggested by EULAR, which, as their paper’s supplementary files highlight, influences outcomes [2]. We therefore recommend that readers look at the supplementary information before drawing conclusions.

Markusse and colleagues propose the presence of three of four characteristics as the definition of PP (erosions, rheumatoid factor/anti-citrullinated protein antibody combination, swollen joint count, elevated Disease Activity Score). In contrast, the definition of PP established by Visser and colleagues in the same trial population (*sic*) uses a different approach, namely C-reactive protein, erosion score and rheumatoid factor/anti-citrullinated protein antibody combination, to determine who had >50 % chance of rapid radiographic progression (≥5 Sharp–van der Heijde Score (SHS) units/year) [5]. The median SHS progression between initial combination therapy and initial monotherapy in PP patients differed only 1.5 SHS units in Markusse and colleagues’ model, but by 3.5 units in that of Visser and colleagues. Of the initial monotherapy patients in Visser’s and Markusse’s models, 64 % and 26 %, respectively, had rapid radiographic progression whereas this proportion was only 12 % and 10 % for initial combination therapy. This observation highlights that the definition of PP used by Visser and colleagues (provided only as supplementary material), in line with other work [6], is much better at identifying a PP population.

The odds of response to initial combination therapy versus initial monotherapy in the PP versus non-PP populations were much higher when using Visser and colleagues’ approach versus Markusse and colleagues’ approach (odds ratio of American College of Rheumatology 20/50/70: 10.0, 9.74, 9.33 vs. 2.72, 5.39, 4.22, respectively). Separation of the Health Assessment Questionnaire score between PP and non-PP patients treated with initial combination therapy is only seen with Visser and colleagues’ definition. This highlights that definition of PP influences the effect of clinical outcomes.

In accordance with the EULAR research agenda [2], we also believe it is important to study what effect patient stratification based on poor prognosis parameters has on clinical outcomes. Alas, we feel that Markusse and colleagues’ study did not address the question appropriately and therefore does not provide a good answer.

## Abbreviations

EULAR: European League Against Rheumatism
PP: Poor prognosis
SHS: Sharp–van der Heijde Score.
